# prevalence of syphilis among pregnant women in China: a systematic review and meta-analysis

**DOI:** 10.1186/s12884-026-08986-6

**Published:** 2026-04-01

**Authors:** Juan Cheng, Bing Song, Yongbin Sun, Huihuang Huang, Tianjun Jiang, Dayu Kuai

**Affiliations:** 1 Senior Department of Infectious Diseases, the Fifth Medical Center of PLA General Hospital, Beijing, China; 2 Department of Gastroenterology, Luhe Hospital, Capital Medical University, Beijing, China

**Keywords:** Syphilis, Prevalence, Pregnant women, Meta-analysis, Systematic review, China

## Abstract

**Objective:**

Syphilis is a serious sexually transmitted infection that can cause severe adverse pregnancy outcomes. This study aimed to estimate the pooled prevalence of syphilis among pregnant women in China using systematic review and meta-analysis.

**Methods:**

This systematic review and meta-analysis were conducted with the PRISMA guidelines. After developing search strategies and registering the study protocol in PROSPERO, we systematically searched Medline, PubMed, Embase, Wiley Online Library, China National Knowledge Infrastructure (CNKI), Wanfang, and Chongqing VIP (CQVIP) for relevant cross-sectional studies conducted in China and published between 2005 and 2024, without language restrictions. The pooled prevalence of syphilis infection was estimated using a random-effects model in STATA software (version 15.0). Results are reported as prevalence estimates with 95% confidence intervals (95% CI), and a p-value < 0.05 was considered statistically significant. Heterogeneity was assessed using the *I²* statistic and Q-test, and publication bias was evaluated using Egger’s and Begg’s tests, trim-and-fill, and funnel plots.

**Results:**

A total of 29 studies involving 8,830,599 pregnant women were included. The overall pooled prevalence of syphilis was 0.46% (95% CI: 0.40%–0.52%). The subgroup analysis revealed regional variations, with the Eastern region showing the highest rate (0.55%), followed by the Western (0.38%) and Central (0.36%) regions. Subgroup analysis by publication period revealed that the prevalence decreased from 0.75% (2005–2010) to 0.34% (2021–2024); however, the decline was not statistically significant (*p* = 0.062).

**Conclusion:**

In recent years, the prevalence of syphilis has shown a declining trend in China, although significant regional disparities remain. Continued attention should be paid to high-risk regional populations.

## Introduction

Syphilis, a sexually transmitted infection caused by *Treponema pallidum*, remains a serious global public health concern. During pregnancy, *Treponema pallidum* infection can cause placental vasculitis and infarction. This leads to adverse outcomes, including spontaneous abortion, stillbirth, intrauterine growth restriction, preterm birth, and low-birth weight. Furthermore, *Treponema pallidum* can cross the placenta and directly infect the fetus, resulting in congenital syphilis. These complications significantly increase perinatal morbidity and mortality [1, 2]. In response, the World Health Organization (WHO) launched the Global Initiative to Eliminate Congenital Syphilis in 2007, later integrating maternal syphilis interventions into existing HIV elimination efforts in 2015 [3, 4].

Since the late 1990s, China has witnessed a significant resurgence in syphilis incidence. In alignment with global goals, a key national strategy has been the integration of syphilis screening into the existing Prevention of Mother-to-Child Transmission (PMTCT) program. This shift marks a move from passive treatment to proactive prevention [5, 6]. Consequently, assessing the prevalence of syphilis among pregnant women under this standardized protocol is crucial. It is vital for monitoring progress and informing future policy.

However, despite over a decade of implementation since the national syphilis control plan was launched in 2010, significant disparities persist in China. The reported infection rate among pregnant women shows high heterogeneity. It remains unclear whether the overall national decline has been uniformed across different economic regions and over time. The persistent burden in some areas indicates an ongoing gap towards the goal of eliminating mother-to-child transmission. This gap has not yet been fully quantified in its spatial and temporal dimensions [7].

To bridge this gap, we conducted a systematic review and meta-analysis to quantify regional inequalities and spatiotemporal heterogeneity in syphilis prevalence among pregnant women in China from 2005 to 2024, with a focus on the post-2010 policy period. By providing an updated national estimate and subgroup analyses, our findings offer a refined scientific basis for targeted prevention and control strategies.

## Methods

### Study protocol and registration

This study was conducted in accordance with the Preferred Reporting Items for Systematic Reviews and Meta-Analyses (PRISMA). The study protocol was prospectively registered with the International Prospective Register of Systematic Reviews (PROSPERO) (registration number: CRD 420251113989).

### Search strategy

A systematic computerized search was performed in the following databases: Medline, PubMed, Embase, Wiley Online Library, China National Knowledge Infrastructure (CNKI), Wanfang, and Chongqing VIP (CQVIP). The search aimed to identify cross-sectional studies on the prevalence of syphilis among pregnant women in China, published between January 1, 2005 and December 31, 2024. The search terms included “syphilis,” “Treponema pallidum,” “pregnant women,” “pregnancy,” “prevalence,” “epidemiology,” “China,” “Chinese,” and their relevant synonyms and variations. For example, the search strategy in PubMed was as follows: ((“syphilis” [MeSH Terms] OR “Treponema pallidum” [MeSH Terms]) AND (“pregnant women” [MeSH Terms] OR “pregnancy” [MeSH Terms]) AND (“China” [MeSH Terms] OR “Chinese”) AND (“prevalence” OR “epidemiology”)).

Database searches were conducted without language restrictions to maximize the identification of relevant literature. However, for practical feasibility and considering that the vast majority of studies on this topic are published in either Chinese or English, this review only considered full-text articles available in these languages.

### Inclusion and exclusion criteria

The inclusion criteria were as follows: (1) study type: observational cross-sectional studies or the baseline survey portion of cohort studies; (2) study population: pregnant women in China aged ≥ 18 years. The lower age limit of 18 years was set to focus on the adult pregnant population and align with the ethical frameworks of the original studies. No upper age limit was applied to ensure the inclusion of all women of childbearing age. While this criterion enhances the representativeness of the sample for estimating the national adult infection rate, it may limit the generalizability of the findings to pregnant adolescents. (3) diagnostic criteria: studies that applied the standard syphilis diagnostic protocol specified in China’s national PMTCT program. This requires a two-step serological testing process: initial screening with a non-treponemal test (e.g., Rapid Plasma Reagin [RPR] or Toluidine Red Unheated Serum Test [TRUST]), followed by confirmation of reactive results with a treponemal test (e.g., Treponema pallidum Particle Agglutination [TPPA] or Enzyme-Linked Immunosorbent Assay [ELISA]). Only studies that defined syphilis cases as positive in both tests were included. (4) outcome measure: Reported the prevalence of syphilis or provided raw data sufficient to calculate the prevalence rate (number of positive cases/total number of individuals tested); (5) timeframe: Studies published in or after 2005.

The exclusion criteria were as follows: (1) duplicate publications; (2) literature for which the full text is unavailable or which contains incomplete data; (3) reviews, commentaries, case reports, conference abstracts, etc.; (4) non-Chinese or non-English publications.

### Study selection and data extraction

Literature screening was conducted independently by two researchers (Song B and Sun YB). To ensure the reliability and consistency of the screening process, we used Cohen’s kappa (κ) coefficient to quantify inter-rater reliability. A κ value greater than 0.80 indicated an excellent agreement. The values of κ = 0.92 for the title/abstract screening stage and κ = 0.88 for the full-text screening stage indicated excellent agreement.

Any discrepancies arising from the comparison of the screening results were resolved through discussion between the two researchers or consultation with a third researcher (Cheng J) until a consensus was reached.

The extracted data included the first author, publication year, study region, sample size, number of syphilis-positive cases, prevalence rate, study design, and diagnostic criteria.

### Risk of bias assessment in included studies

Two authors (Song B and Sun YB) independently assessed and cross-checked the methodological quality of the included studies using The Joanna Briggs Institute (JBI) Critical Appraisal Checklist for Analytical Cross-Sectional Studies [8]. Any discrepancies were discussed and analyzed during group meetings until a consensus was reached, thereby ensuring the reliability of the results. This tool consists of nine items which cover multiple key aspects of study design, including the sample frame, sampling method, sample size, description of the study setting, completeness of data analysis, validity of condition identification and measurement, appropriateness of statistical analysis and response rate.

The overall quality scores were high, ranging from 7 to 9 (out of 9). Common shortcomings included inadequate description of sampling methods in 22 studies and unclear reporting of non-response rates in 6 studies.

### Statistical analysis

For each included study, the prevalence rate and 95% confidence interval (CI) were calculated based on the number of positive cases and total sample size. Heterogeneity among studies was assessed using the *I²* statistic, with an *I²* value greater than 50% considered indicative of significant heterogeneity. Given the anticipated and subsequently confirmed substantial heterogeneity across studies (*I²* > 90%), a random-effects model was employed to calculate the pooled prevalence rate and 95% confidence interval (CI). This model accounts for variability in true effect sizes across studies, providing a more conservative and generalizable estimate than the fixed-effect model.

To explore the sources of heterogeneity, subgroup analyses were planned based on: (1) geographical region: Studies were stratified according to the three major economic-geographic regions of China (Eastern, Central, and Western). This classification is based on the strategic framework first formally proposed in The CPC Central Committee’s Proposal on Formulating the Seventh Five-Year Plan for National Economic and Social Development, which reflects the national-level regional development strategy. This framework has since been widely adopted in socioeconomic and public health research to account for systematic disparities in development and resource distribution across the country. (2) publication period: Studies were grouped into 5-year intervals for subgroup analyses. To ensure that each interval was complete at the time of data collection, the most recent period was defined as 2021–2024.

Sensitivity analyses were performed to evaluate the robustness of the pooled results by excluding studies with the highest prevalence rates and those with lower-quality scores. Publication bias was assessed through visual inspection of funnel plots supplemented by Egger’s and Begg’s tests. To further quantify the potential impact of funnel plot asymmetry, the trim-and-fill method was applied to impute potentially missing studies. All analyses were conducted using STATA software (version 15.0) with the meta package, and a two-sided p-value < 0.05 was considered statistically significant.

## Results

### Literature screening process and results

**Fig. 1:**
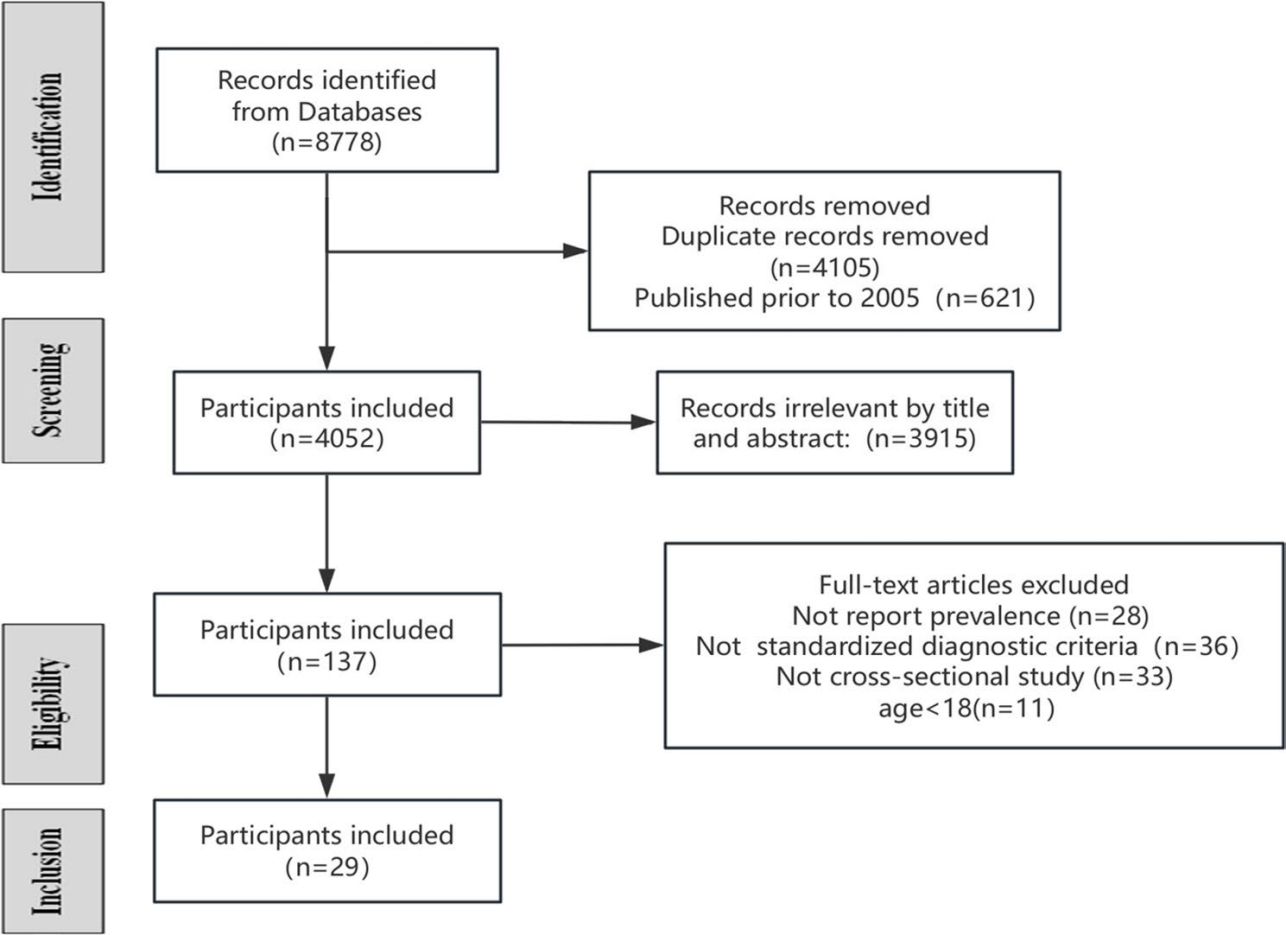
PRISMA flow chart of study selection, according to PRISMA guidelines

Figure 1 presents the PRISMA flow diagram of the study selection process. Our initial identification yielded 8,778 records. After removing 4,105 duplicates and 621 pre-2005 publications, 4,052 titles and abstracts were screened. Of these, 3,915 were excluded as irrelevant, leaving 137 full-text articles for further assessment. A further 108 articles were excluded for the following reasons: not reporting prevalence (*n* = 28), lacking standardized diagnostic criteria (*n* = 36), not being cross-sectional (*n* = 33), or including participants < 18 years (*n* = 11). Consequently, 29 studies were included in the review. (Fig. 1)

### Basic characteristics and quality assessment of included studies

The basic characteristics of the included studies are presented in Table 1. The 29 studies covered multiple provinces in Eastern, Central, and Western China. The sample sizes of individual studies ranged from 1,074 to 4,458,696. All studies clearly described the serological diagnostic methods for syphilis. According to the JBI appraisal tool, the quality scores of the included studies ranged from 7 to 9 (out of a total score of 9), indicating an overall high methodological quality.

**Table 1:** General characteristics of the studies included in the systematic review and meta-analysis of the prevalence of syphilis in pregnant women in China

Author and year of publication	Region, location	Sample size, *n*	Prevalence (%)	JBI Quality Score (1 to 9)
He Y (2009) [9]	Guangzhou	17,943	0.6186	8
Shao MM (2009) [10]	Shanxi	15,016	0.2264	8
Wei H (2010) [11]	Guangxi	17,524	1.0899	8
Liu Q(2010) [12]	Hainan	48,602	0.5843	8
Zhong DM (2010) [13]	Guangzhou	10,754	2.2038	8
Yang LG (2013) [14]	Guangdong	27,150	0.3904	9
Lin GL (2013) [15]	Guangdong	3649	0.3563	7
Yang JL (2013) [16]	Zhejiang	3854	1.5049	7
Feng L (2013) [17]	Chongqing	1743	0.7458	7
Qin JB (2014) [18]	Shenzhen	279,334	0.3	9
Jiang YY (2015) [19]	Guangxi	9365	0.2136	7
Peng Y (2015) [20]	Shandong	11,661	0.1458	8
Zhang XH (2016) [21]	Zhejiang	1,338,739	0.3148	9
Xie Q (2016) [22]	Hubei	2200	0.3636	7
Zhong LC (2017) [23]	Guangdong	12,704	0.4565	8
Li W (2017) [24]	Tianjin	139,401	0.8587	7
Xu X (2017) [25]	Guangzhou	1074	1.1173	7
Yao JS (2019) [26]	Neimeng	10,291	0.2818	7
Li XH (2020) [27]	Neimeng	53,710	0.4338	8
Zhang X (2022) [28]	Beijing	144,690	0.132	8
Gao J (2023) [29]	Hunan	4,458,696	0.3189	9
Liu HH (2023) [30]	Guangzhou	694,894	0.2002	7
Shi LG (2023) [31]	Jiangsu	1,272,810	0.2069	8
Wang X (2023) [32]	Henan	22,458	0.0935	8
Sun YY (2023) [33]	Henan	52,850	0.4125	8
Xie YR (2023) [34]	Zhejiang	11,260	1.8472	7
Patil S (2024) [35]	Guangxi	54,048	0.0999	9
Sun MT (2024) [36]	Hunan	34,074	0.5576	9
Shang XL (2024) [37]	Henan	80,105	0.5093	8

### Pooled prevalence of syphilis

**Fig. 2:**
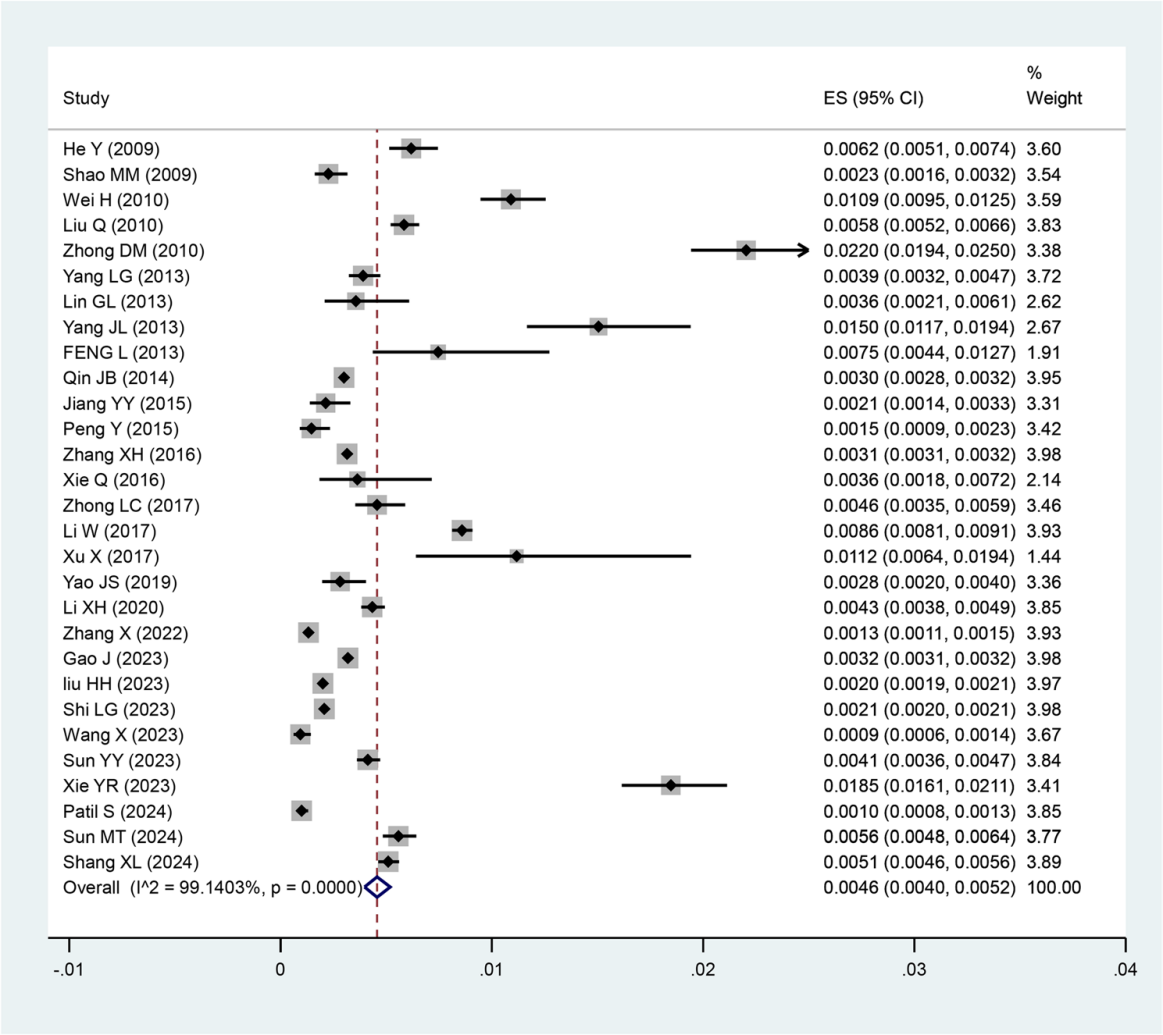
The combined overall prevalence of syphilis among pregnant women in China: 0.46% (95% CI: 0.40% − 0.52%)

The estimated prevalence values, weights, and combined results of each study are shown in Fig. 2. The overall co-prevalence of syphilis among pregnant Chinese women was 0.46% (95% CI: 0.40%-0.52%). This analysis detected extremely high heterogeneity (*I²* = 99.1403%, *p* < 0.05).

### Subgroup analysis

**Fig. 3:**
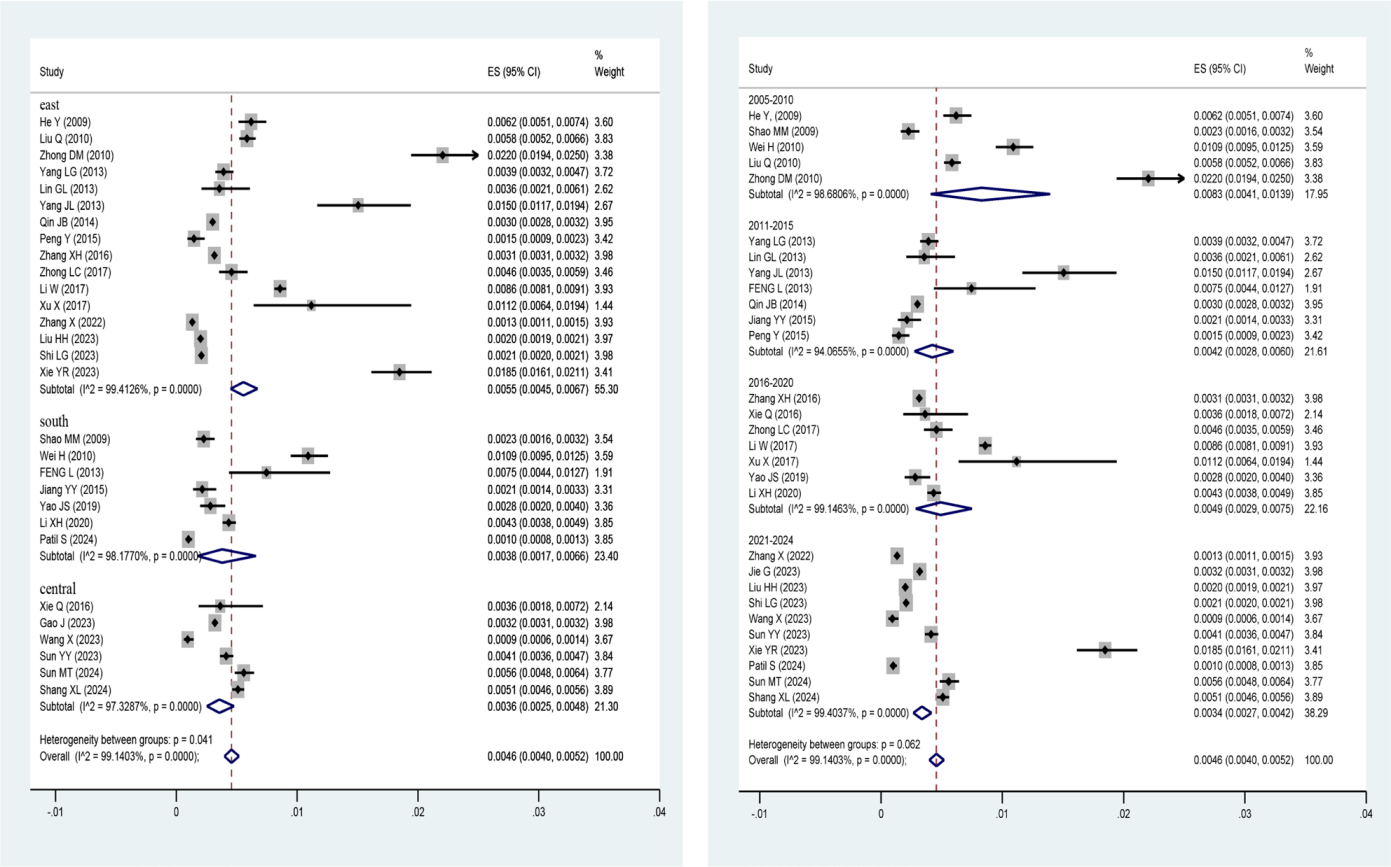
Subgroup analysis by geographical region and time period of publication

By geographical region (left side of Fig. 3), the Eastern region had the highest pooled prevalence at 0.55% (95% CI: 0.45%-0.67%), followed by the Western region at 0.38% (95% CI: 0.17%-0.66%). The Central region had the lowest prevalence at 0.36% (95% CI: 0.25%-0.48%). The differences between the groups were statistically significant (*p* = 0.041).

By publication period (right side of Fig. 3), studies published between 2005 and 2010 reported the highest pooled prevalence of 0.75% (95% CI: 0.60%–0.90%). The infection rate significantly decreased to 0.49% (95% CI: 0.40%–0.58%) during 2011–2015. The prevalence remained at 0.47% (95% CI: 0.38%–0.56%) during 2016–2020. Studies published between 2021 and 2024 indicated a low prevalence of 0.34% (95% CI: 0.28%–0.40%).

### Sensitivity analyses

**Fig. 4:**
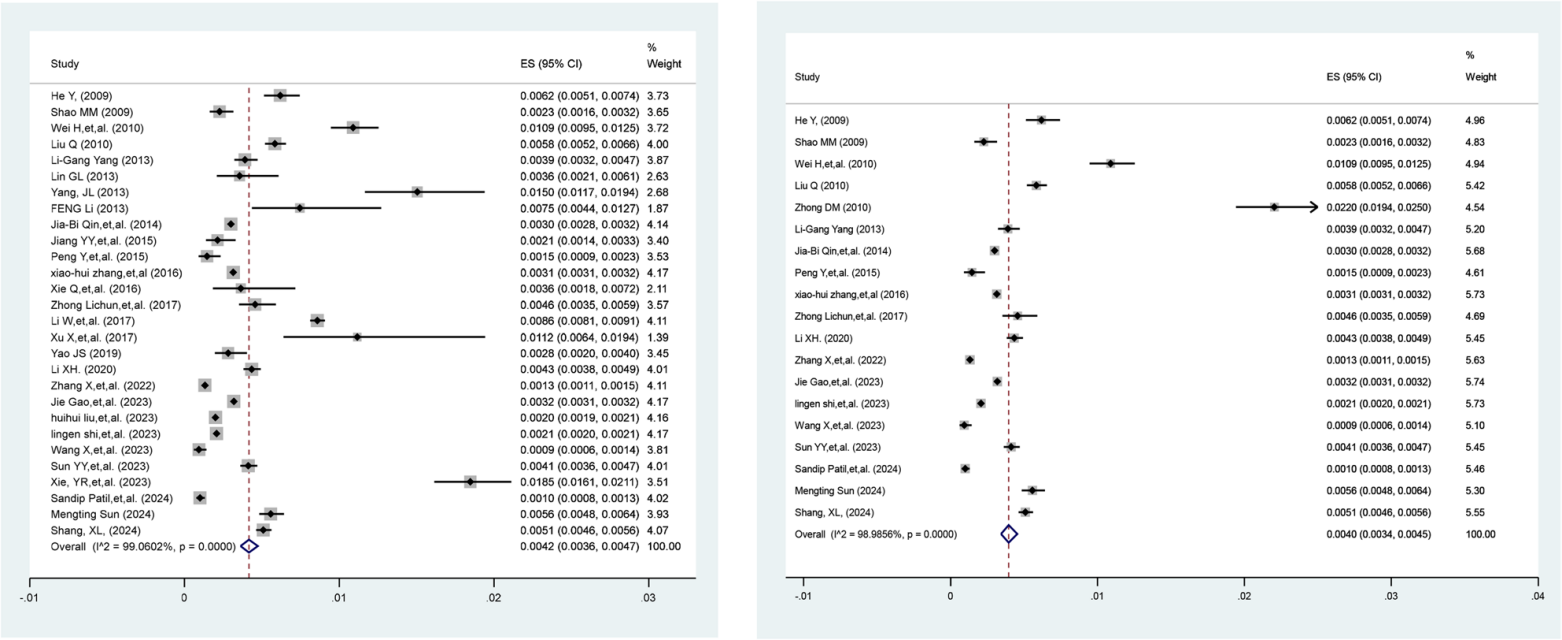
Sensitivity analyses by geographical region and time period of publication

We conducted two sensitivity analyses to assess the stability of the combined results. We excluded the study with the highest prevalence rate [13]. Upon reanalysis, the pooled prevalence was 0.42% (95% CI: 0.36%-0.47%) (left side of Fig. 4). We excluded all studies with a literature quality score (JBI score) of 7, retaining only those with a score of ≥ 8 for inclusion in the meta-analysis. The results showed a pooled prevalence of 0.40% (95% CI: 0.34%-0.45%), indicating robust results (right side of Fig. 4).

### Publication bias

**Fig. 5:**
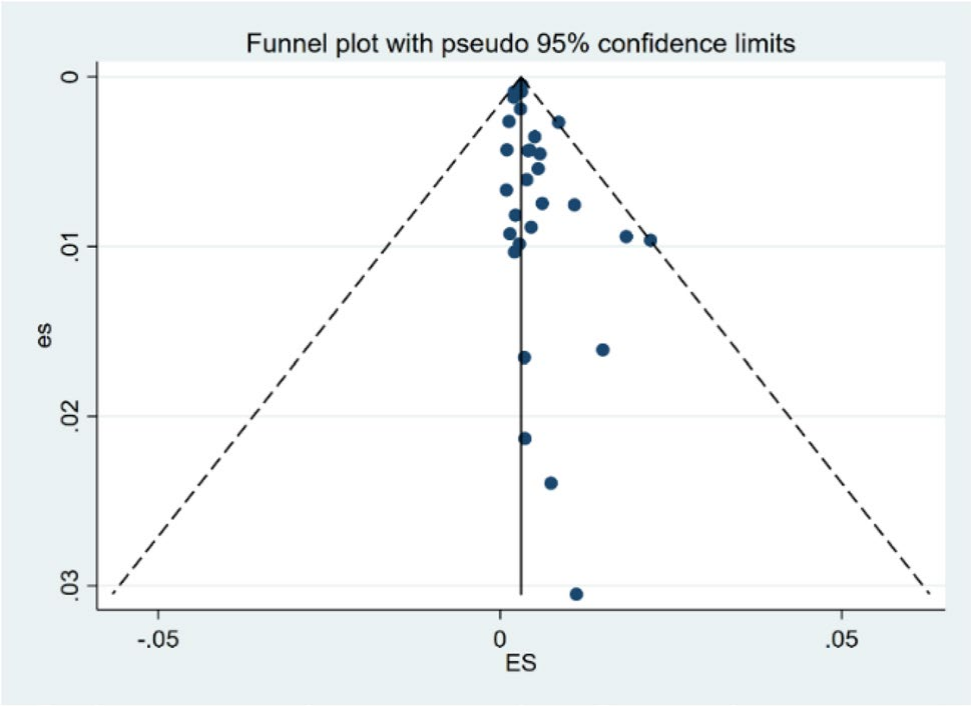
Funnel plot of the studies included in the systematic review

**Fig. 6:**
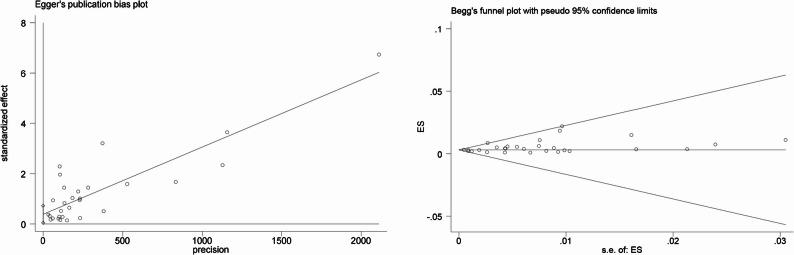
Egger’s and Begg’s tests

**Fig. 7:**
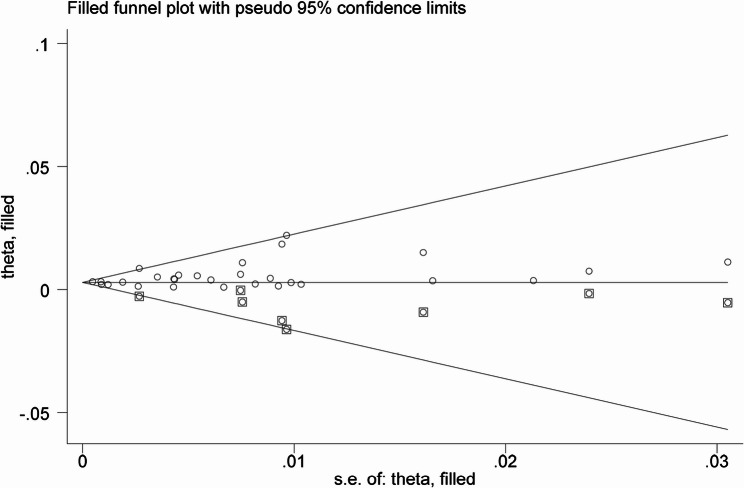
Funnel plot with trim-and-fill adjustment

The funnel plot showed a degree of asymmetry (Fig. 5). The results of the quantitative test indicated that Egger’s test suggested a statistically significant publication bias (t = 2.29, *p* = 0.03) (Table 2). However, Begg’s rank correlation test did not find evidence of significant publication bias (z = 1.07, *p* = 0.285) (Fig. 6). The trim-and-fill method imputed eight theoretically missing studies to achieve funnel plot symmetry. The adjusted pooled prevalence after imputation was 0.30% (95% CI: 0.20%–0.40%), which was slightly lower than the original estimate of 0.46% (Fig. 7).

**Table 2:** Egger’s test

Std Eff	Coef.	Std. Err.	t	*P*-value	95% CI lower	95% CI upper
slope	0.0026724	0.0003009	8.88	< 0.001	0.0020549	0.0032899
bias	0.3809673	0.1660629	2.29	0.030	0.0402343	0.7217003

Egger’s test is used to assess the publication bias

*CI* Confidence interval

## Discussion

This study synthesized data from 29 studies across different regions of China, involving 8,830,599 pregnant women, to comprehensively assess the infection rate and spatiotemporal distribution of syphilis among pregnant women in China. The main findings were as follows: the overall pooled prevalence of syphilis among pregnant women in China was 0.46%; there were significant regional differences in prevalence, with the economically developed Eastern region having a significantly higher rate than the Central and Western regions; temporally, the prevalence significantly decreased from a high level after 2011, with recent data suggesting a possible further decline.

The syphilis infection rate rate of 0.46% among pregnant women in China is at a low epidemic level globally, lower than the global pooled prevalence (0.8%) [38], and significantly lower than that in high-burden regions such as sub-Saharan Africa (> 2%) and Brazil (1.79%) [39, 40]. However, compared to developed countries in Western Europe and North America (typically < 0.1%) [38], China still lags. This success underscores the effectiveness of integrating syphilis screening into the national PMTCT program. The integration has markedly improved early detection and intervention rates. Yet, the gap with developed nations highlights the ongoing need for sustained efforts. Further refinement of prevention strategies is required to move closer to the goal of eliminating mother-to-child transmission.

The substantial regional disparities identified in this study merit further investigation. The highest disease burden in the economically developed eastern region may be attributable to several factors [41]. First, as the country’s primary economic hub, the eastern region attracts many internal migrants. Systematic reviews have confirmed that this population faces a significantly higher risk of sexually transmitted infections than the general population; the infection rate of syphilis among migrants is 0.69% (95% CI: 0.57%-0.84%), approximately 1.9 times that of the general population (OR = 1.9, 95% CI: 1.1-3.0) [42]. Second, disparities in healthcare service accessibility persist across regions. The maternal mortality ratio in the western region is 118% higher than that in the eastern region (2.18, 1.44–3.28), while the central region is 41% higher (1.41, 0.99–2.01) [43]. This inequitable distribution of health resources may affect the coverage, quality, and reporting of syphilis screening and intervention. These findings suggest differentiated strategies based on regional characteristics: in the eastern region, leveraging relatively well-established health service systems to promote high-quality interventions and precise epidemic control; in central and western regions, continuing to strengthen primary healthcare service capacity and surveillance networks.

The temporal trend analysis showed that the high prevalence period from 2005 to 2010 reflected the severe challenges faced at that time. The “turning point” and the subsequent continuous decline after 2011 aligned with the issuance and comprehensive implementation of the China Syphilis Prevention and Control Plan (2010–2020). The continued decline in recent years suggests that interventions have evolved beyond short-term campaigns into sustainable, long-term mechanisms. However, it is important to note that the observed decline in recent years (2021–2024 publications) should be interpreted with caution, as these studies likely reflect data collected in earlier years (see Limitations).

Some included studies reported prevalence estimates substantially higher than the overall level, including a study from Guangzhou that reported 2.20% [13]. These outliers may be attributable to localized outbreaks, sampling bias from studies conducted in large general hospitals overrepresenting women with pregnancy complications, or inadvertent inclusion of higher proportions of high-risk populations. Notably, sensitivity analysis excluding extreme values yielded a pooled prevalence of 0.42% (95% CI: 0.36%-0.47%), highly consistent with the original result (0.46%). This suggests that individual data discrepancies do not undermine the robustness of the core conclusions.

Regarding publication bias, the discrepancy between Egger’s test (significant) and Begg’s test (non-significant) is not uncommon, as Egger’s test is generally considered more sensitive. The trim-and-fill adjustment suggested a potential slight overestimation, although the adjusted estimate (0.30%) remained consistent with our primary finding, reinforcing the robustness of the main conclusions.

## Limitations

This study has several limitations that should be considered when interpreting the findings.

First, this study applied an age inclusion criterion of ≥ 18 years, primarily to focus on the adult pregnant population and comply with the ethical review requirements of the original studies. Consequently, pregnant adolescents (< 18 years) were excluded. Adolescent populations differ systematically from pregnant adult women in terms of sexual behavior patterns, healthcare access, and infection risks. Existing evidence consistently identifies adolescents as a higher-risk group for sexually transmitted infections [44]; their exclusion may therefore lead to a slight underestimation of the overall syphilis prevalence among pregnant women and limit the generalizability of the findings to the full age spectrum of pregnant populations.

Second, substantial heterogeneity was observed across included studies. Although subgroup analyses partially explained sources of variation, unmeasured confounding factors-such as urban-rural disparities, ethnic composition, and high-risk occupational groups-remained uncontrolled. Additionally, funnel plot asymmetry and significant Egger’s test suggested potential publication bias, possibly attributable to small-study effects, language bias (inclusion of only Chinese and English publications), and reporting bias. However, trim-and-fill adjustment (0.30% vs. original 0.46%) indicated that any overestimation was minor and overall conclusions remain robust. Furthermore, most included studies were conducted in urban hospital antenatal clinics, limiting representativeness for rural areas, migrant populations, and ethnic minority groups.

Third, certain limitations exist concerning the diagnostic criteria. Although all included studies employed the standard ‘dual-positivity’ serological criteria, this criterion may occasionally lack sensitivity, particularly during the early serological window period of primary infection. Measurement bias could also arise from differences in specific test kits, operational procedures, or positivity thresholds. False negatives may occur during the serological window period, in very late-stage infections, or due to delayed serological responses in immunosuppressed individuals, potentially leading to under-ascertainment of cases.

Fourth, the temporal trends analysis has limitations. The synthesized data consist of discrete cross-sectional data points that do not meet the continuity assumptions required for time-series analysis; therefore, the interpretation of long-term trends should be approached cautiously. Moreover, using “publication period” as a proxy for the “data collection period” introduces variable time lag, meaning estimates for the most recent publication period (2021–2024) likely reflect data collected earlier and may not fully represent real-time epidemic dynamics. Additionally, the limited number of studies in this recent subgroup means that the stability of the prevalence estimates for these periods requires further validation by future research.

## Conclusion

This meta-analysis indicates that the prevalence of syphilis among pregnant women in China is generally low and has shown a significantly declining trend since 2010, reflecting the effectiveness of national STD prevention and control and PMTCT programs. However, the relatively high prevalence in the eastern region and persistent regional disparities suggest that prevention and control efforts must be reinforced. It is essential to move beyond uniform national policies toward targeted interventions tailored to high-burden populations and regions, ensuring efficient resource allocation to address persistent disparities and achieve the goal of eliminating mother-to-child transmission.

Recommendations for the future are as follows: (1) continue to consolidate and strengthen nationwide maternal syphilis screening and comprehensive intervention measures to ensure the accessibility and quality of services; (2) prioritize the eastern region and high-prevalence provinces, implementing targeted health education and behavioral interventions for key populations such as migrant populations; (3) enhance syphilis surveillance and data reporting systems-including the integration of innovative digital health technologies such as smart systems for real-time data reporting and quality monitoring-to improve the efficiency and equity of the prevention of mother-to-child transmission program, and to enable more precise, data-driven resource allocation and strategy adjustment [45].

## Data Availability

All data are included in this published article. The full search strategies for all databases are provided in the Supplementary Materials.
